# Human Impacts and Climate Change Influence Nestedness and Modularity in Food-Web and Mutualistic Networks

**DOI:** 10.1371/journal.pone.0157929

**Published:** 2016-06-20

**Authors:** Kazuhiro Takemoto, Kosuke Kajihara

**Affiliations:** Department of Bioscience and Bioinformatics, Kyushu Institute of Technology, Iizuka Fukuoka, Japan; Swedish University of Agricultural Sciences, SWEDEN

## Abstract

Theoretical studies have indicated that nestedness and modularity—non-random structural patterns of ecological networks—influence the stability of ecosystems against perturbations; as such, climate change and human activity, as well as other sources of environmental perturbations, affect the nestedness and modularity of ecological networks. However, the effects of climate change and human activities on ecological networks are poorly understood. Here, we used a spatial analysis approach to examine the effects of climate change and human activities on the structural patterns of food webs and mutualistic networks, and found that ecological network structure is globally affected by climate change and human impacts, in addition to current climate. In pollination networks, for instance, nestedness increased and modularity decreased in response to increased human impacts. Modularity in seed-dispersal networks decreased with temperature change (i.e., warming), whereas food web nestedness increased and modularity declined in response to global warming. Although our findings are preliminary owing to data-analysis limitations, they enhance our understanding of the effects of environmental change on ecological communities.

## Introduction

Many species interact with one another via antagonistic (e.g., prey–predator) and mutualistic (e.g., plant–pollinator) relationships, and they compose ecological communities that are often represented as networks [1,2], or *ecological networks*. Ecological networks are important not only in the context of basic scientific research (e.g., structure–stability relationships [1,3–6]), but also in the context of applied ecology (e.g., biodiversity maintenance, environmental assessment [1,3,4]); thus, ecological networks have been studied from a complex network perspective for decades, inspired by the development of network science [7,8]. In addition, a significant amount of data on real-world ecological networks have been collected and are available from such sources as GlobalWeb [9], the Interaction Web DataBase, and the Web-of-Life Database, among others (see also Materials and Methods).

Empirical ecological networks are known to display two non-random structural patterns. One is nested architecture (*nestedness*) [10], a hierarchical structure in which the interaction pairs of a specialist species are included in those of another (generalist) species. The other is modular structure (*modularity*) [11], a compartmentalized structure in which a number of dense sub-networks (modules) are weakly interconnected. Modularity is also a significant property of biological systems [12], such as signaling [13] and metabolic [14–16] networks. Although these two structural patterns generally correlate with each other, the direction and magnitude of the correlation differ depending on network type and the level of connectedness (connectance or graph density); thus, these structural patterns provide complementary information on how interactions are organized in communities [17]. The degree of nestedness and modularity vary between antagonistic networks (e.g., food webs) and mutualistic networks (e.g., plant–pollinator networks); the modularity of mutualistic networks is typically lower than that of antagonistic networks, whereas the nestedness of mutualistic networks is generally higher than that of antagonistic networks [5,10]. Furthermore, studies have shown that nestedness and modularity influence ecosystem dynamics; in particular, nestedness plays important roles in increasing mutualistic-network stability [18–22]. Moreover, Thébault and Fontaine [5] demonstrated that both nestedness and modularity influence ecosystem stability (i.e., persistence and resilience against perturbations). The contributions of nestedness and modularity to ecosystem stability differ between mutualistic and antagonistic networks, in that increasing nestedness and/or decreasing modularity enhance the stability of mutualistic networks but reduce the stability of antagonistic networks (i.e., food webs).

In this context, the effects of environmental or external factors on ecological networks are also important. Given that environmental factors can be sources of perturbation (e.g., rainfall, seasonal variation of climate), it would be expected that ecological networks have an optimal structure that maximizes ecosystem stability against such perturbations. Thus, nestedness and modularity change in response to environmental perturbations because they largely determine ecosystem stability [5]. The importance of environmental factors are often discussed in the context of ecosystem stability [23,24]; many previous studies [25,26] have focused on the association between the environment and ecological network structure, with several focusing specifically on the influence of the environment on nestedness/modularity. Several studies [27–30] have reported the relationship between climatic parameters and nestedness and modularity in mutualistic networks and food webs; for example, nestedness in pollination networks was found to decrease with annual precipitation [27], whereas modularity in seed-dispersal networks increased with temperature seasonality [28], and a positive correlation between modularity and precipitation seasonality was observed in food webs [29]. Previously [29], we demonstrated that climate seasonality affects ecological networks, and that the type of climatic seasonality influencing network structure differs among ecosystems; for example, network properties in freshwater ecosystems were mainly affected by rainfall seasonality but primarily by temperature seasonality in terrestrial ecosystems.

Climate change and human activities are also major sources of environmental perturbations, and given that nestedness and modularity are expected to change in response to environment perturbations, climate change and human impacts are thus also expected to affect ecological network structure. Dalsgaard et al. [31], for instance, reported that modularity and nestedness in pollination networks correlated with the historical rate of warming, and Sebastián-González et al. [32] demonstrated that modularity declined and nestedness increased in seed-dispersal networks in response to human impacts (e.g., human population density, land use, infrastructure development, and so forth). Such results imply that mutualistic networks are flexible and can change in response to climate change and human activities in order to improve ecosystem stability.

Despite this pioneering research, the impacts of climate change and human activities on ecological networks remain poorly understood, especially with respect to the human impact on pollination networks. Moreover, macro-ecological studies [28,31,32] have overwhelmingly focused on the impacts of climate change and human activities on mutualistic networks (i.e., pollination and seed-dispersal networks), and not food webs, for which there has been comparatively little research (e.g., the effect of human activities on prey–predator interactions at a local scale [33] and over a broader marine region [34]).

We therefore constructed a larger dataset of ecological networks—including food webs, pollination networks, and seed-dispersal networks—and used spatial analysis to investigate the effects of climate change and human activities on these networks.

## Results

### Distribution of ecological networks

We identified 126 food-web (S1 Table), 62 pollination (S2 Table), and 30 seed-dispersal (S3 Table) networks for use in this analysis. The food-web and mutualistic networks (i.e., pollination and seed-dispersal networks) represented binary versions of directed and bipartite networks, respectively.

**Fig 1:**
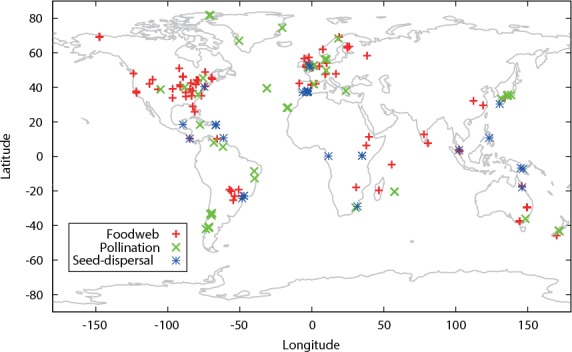
Locations of the ecological network sites included in this study. Symbols indicate observed ecological networks.

Data for the food-web, pollination, and seed-dispersal networks were collected from around the world (Fig 1). It must be noted, however, that the data was likely to be somewhat biased; for one, research on ecological networks tends to derive from several specific countries and not others (see [29] for details), a sampling bias that suggests spatial autocorrelation in the data. As such, we adopted a spatial eigenvector mapping (SEVM) modeling approach during data analysis (see the following sections) in order to remove any inherent spatial autocorrelation, in addition to application of ordinary least squares (OLS) regression analysis (see Materials and Methods).

### Nestedness increased and modularity decreased in pollination networks in response to human impacts

Although pollination and seed-dispersal networks fundamentally differ in many ways, both networks consist of mutually beneficial relationships (or +/+ interactions) and are thus categorized as mutualistic networks; as such, they would be expected to exhibit many similar properties, at least in the context of ecosystem-stability theory (e.g., [3,5]).

**Fig 2:**
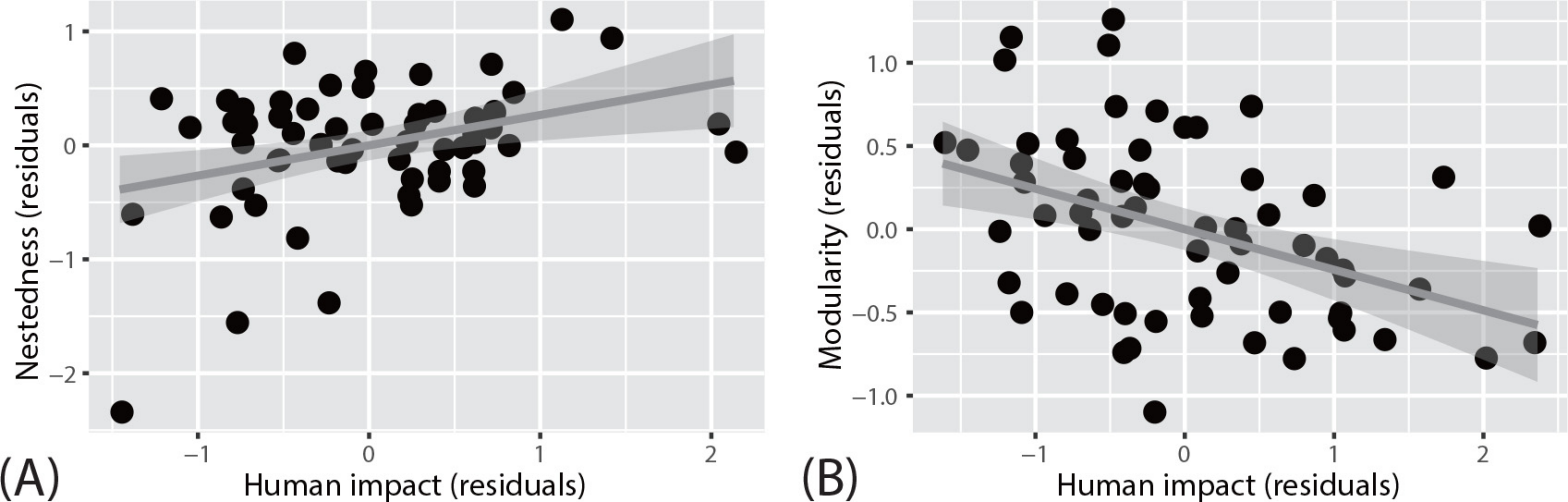
Scatter plots of a network parameter (residuals) versus human impact (residuals) in pollination networks. Nestedness (A) and modularity (B).

We found a positive association between nestedness and human impacts (Table 1 and Fig 2A) but a negative association between modularity and human impacts (Table 2 and Fig 2B). In the OLS regression analysis, the best model indicated that nestedness increases with (historical) temperature-change velocity based on the absolute difference between current and Last Glacial Maximum climate conditions (see Materials and Methods); however, the averaged model indicated that such an association was limited. Moreover, spatial autocorrelation analysis (*p* < 0.01, using the Moran’s test; Table 1) suggested that the observed association is in fact merely an artifact; no association between the rate of temperature change rate and nestedness/modularity was detected when spatial dependency was removed from the regression residuals (i.e., when an SEVM modeling approach is applied).

**Table 1:** Influences of explanatory variables on nestedness in pollination networks.

Variables	OLS				SEVM			
	Estimate (full)	Estimate (best)	Estimate (averaged)	Importance	Estimate (full)	Estimate (best)	Estimate (averaged)	Importance
**#Species**	0.708 (<0.01)	0.678 (<0.01)	0.650 (<0.01)	1.00	0.732 (<0.01)	0.737 (<0.01)	0.722 (<0.01)	1.00
**Elevation**	-0.201 (0.25)		-0.196 (0.26)	0.40	-0.375 (0.01)	-0.441 (<0.01)	-0.429 (<0.01)	0.98
***T***_**mean**_	-0.517 (0.02)	-0.415 (0.02)	-0.391 (0.08)	0.63	-0.585 (<0.01)	-0.630 (<0.01)	-0.674 (<0.01)	0.98
***T***_**seasonality**_	-0.624 (<0.01)	-0.505 (0.01)	-0.449 (0.05)	0.82	-0.560 (<0.01)	-0.595 (<0.01)	-0.597 (<0.01)	0.98
***P***_**ann**_	-0.087 (0.52)		-0.190 (0.16)	0.47	-0.233 (0.07)	-0.237 (0.05)	-0.237 (0.06)	0.64
***P***_**seasonality**_	0.216 (0.19)	0.253 (0.08)	0.216 (0.21)	0.44	0.475 (<0.01)	0.433 (<0.01)	0.473 (<0.01)	0.99
**Human impact**	0.224 (0.10)	0.236 (0.07)	0.217 (0.14)	0.50	0.308 (<0.01)	0.313 (<0.01)	0.333 (<0.01)	0.98
***T***_**velocity**_	0.237 (0.21)	0.389 (0.01)	0.296 (0.09)	0.62	0.134 (0.39)		0.146 (0.36)	0.21
***P***_**velocity**_	0.077 (0.55)		0.068 (0.63)	0.26	-0.037 (0.72)		-0.010 (0.93)	0.13
**Moran’s *I***	0.44 (<0.01)	0.41 (<0.01)			-0.22 (0.34)	-0.20 (0.34)		
***R***^**2**^	0.47 (<0.01)	0.45 (<0.01)			0.77 (<0.01)	0.77 (<0.01)		

*T*_mean_ and *T*_seasonality_ indicate mean annual temperature and temperature seasonality, respectively; *P*_ann_ and *P*_seasonality_ represent annual precipitation and precipitation seasonality, respectively; *T*_velocity_ and *P*_velocity_ represent mean temperature-change velocity and precipitation-change velocity, respectively. The estimates in the full, best, and averaged models based on the ordinary least squared (OLS) regression and spatial eigenvector mapping (SEVM) modeling approach (see Materials and Methods) are shown. *R*^2^ denotes the coefficient of determinations for full/best models based on the OLS regression and SEVM modeling. Values in brackets are the associated *P*-values.

**Table 2:** Influences of explanatory variables on modularity in pollination networks.

Variables	OLS				SEVM			
	Estimate (full)	Estimate (best)	Estimate (averaged)	Importance	Estimate (full)	Estimate (best)	Estimate (averaged)	Importance
**#Species**	0.683 (<0.01)	0.700 (<0.01)	0.714 (<0.01)	1.00	0.805 (<0.01)	0.845 (<0.01)	0.824 (<0.01)	1.00
**Elevation**	0.200 (0.17)		0.138 (0.22)	0.42	0.208 (0.09)		0.139 (0.16)	0.46
***T***_**mean**_	0.137 (0.46)		0.115 (0.38)	0.35	-0.020 (0.92)		-0.008 (0.97)	0.25
***T***_**seasonality**_	0.031 (0.86)		-0.075 (0.56)	0.29	-0.398 (0.07)	-0.376 (<0.01)	-0.379 (<0.01)	0.94
***P***_**ann**_	0.192 (0.10)	0.176 (0.04)	0.188 (0.06)	0.66	0.115 (0.23)		0.119 (0.19)	0.38
***P***_**seasonality**_	0.053 (0.70)		0.067 (0.56)	0.27	-0.083 (0.45)		-0.084 (0.40)	0.25
**Human impact**	-0.217 (0.06)	-0.205 (0.02)	-0.209 (0.04)	0.77	-0.209 (0.03)	-0.240 (<0.01)	-0.225 (0.01)	0.92
***T***_**velocity**_	0.118 (0.45)		-0.024 (0.86)	0.26	0.085 (0.49)		0.036 (0.76)	0.20
***P***_**velocity**_	0.034 (0.75)		0.031 (0.74)	0.23	0.093 (0.32)		0.068 (0.42)	0.24
**Moran’s *I***	0.16 (0.04)	0.17 (0.08)			-0.23 (0.51)	-0.24 (0.72)		
***R***^**2**^	0.63 (<0.01)	0.60 (<0.01)			0.81 (<0.01)	0.78 (<0.01)		

See Table 1 for description of table elements.

In a previous study [31], increasing nestedness and declining modularity owing to temperature-change velocity were only observed for mainland pollination networks; such associations were not conclusive for island pollination networks or the global dataset. As the results presented here were based on global datasets, it is possible that the limited effect of temperature-change velocity on nestedness and modularity is because we differentiated between mainland and island networks. Therefore, we performed similar analyses for both mainland and island networks for reasons of comparison. We found a positive correlation between nestedness and human impacts in mainland networks (S4 Table), but not in island networks (S5 Table), whereas modularity was negatively associated with human impacts in both mainland (S6 Table) and island (S7 Table) networks. On the other hand, no relationship was detected between temperature-change velocity and nestedness/modularity in either mainland or island networks (S4–S7 Tables). These results suggest that nestedness and modularity in pollination networks are influenced by human impacts rather than temperature-change velocity, a discrepancy that may exist for several reasons (see Discussion).

Several geographical and climatic parameters were also factors in determining nestedness and modularity; nestedness decreased with elevation, mean annual temperature, and temperature seasonality, and increased with precipitation seasonality, whereas modularity decreased with temperature seasonality. These results are somewhat consistent with the results of other studies; for example, a negative association between nestedness and temperature seasonality has been previously reported [29].

### Nestedness increased and modularity decreased in food webs in response to temperature-change velocity

Food webs are categorized into antagonistic networks, which differ significantly from mutualistic networks. We focused on the results obtained from the SEVM modeling approach because spatial autocorrelation was completed in the OLS regression models (*p* < 0.01, Moran’s test; Tables 3 and 4).

**Table 3:** Influences of explanatory variables on nestedness in food webs.

Variables	OLS				SEVM			
	Estimate (full)	Estimate (best)	Estimate (averaged)	Importance	Estimate (full)	Estimate (best)	Estimate (averaged)	Importance
**#Species**	-0.017 (0.84)		-0.035 (0.68)	0.27	-0.033 (0.67)		-0.042 (0.58)	0.27
**Elevation**	0.103 (0.21)		0.087 (0.29)	0.38	0.048 (0.51)		0.033 (0.65)	0.25
***T***_**mean**_	0.050 (0.75)		0.084 (0.62)	0.32	0.220 (0.13)	0.219 (0.03)	0.285 (0.02)	0.77
***T***_**seasonality**_	-0.308 (0.09)	-0.329 (<0.01)	-0.327 (<0.01)	0.92	-0.155 (0.34)	-0.217 (0.05)	-0.255 (0.07)	0.62
***P***_**ann**_	0.239 (0.01)	0.268 (<0.01)	0.254 (<0.01)	0.97	0.076 (0.37)		0.099 (0.25)	0.39
***P***_**seasonality**_	-0.208 (0.03)	-0.143 (0.05)	-0.171 (0.04)	0.76	-0.067 (0.48)		-0.058 (0.51)	0.28
**Human impact**	0.072 (0.42)		0.061 (0.48)	0.31	0.082 (0.32)		0.101 (0.20)	0.43
***T***_**velocity**_	0.555 (<0.01)	0.595 (<0.01)	0.580 (<0.01)	1.00	0.336 (<0.01)	0.356 (<0.01)	0.331 (<0.01)	0.99
***P***_**velocity**_	0.071 (0.57)		0.047 (0.72)	0.30	-0.019 (0.87)		-0.054 (0.61)	0.31
**Moran’s *I***	0.25 (<0.01)	0.29 (<0.01)			-0.12 (0.51)	-0.12 (0.61)		
***R***^**2**^	0.40 (<0.01)	0.39 (<0.01)			0.56 (<0.01)	0.55 (<0.01)		

See Table 1 for description of table elements.

**Table 4:** Influences of explanatory variables on modularity in food webs.

Variables	OLS				SEVM			
	Estimate (full)	Estimate (best)	Estimate (averaged)	Importance	Estimate (full)	Estimate (best)	Estimate (averaged)	Importance
**#Species**	0.488 (<0.01)	0.513 (<0.01)	0.528 (<0.01)	1.00	0.518 (<0.01)	0.527 (<0.01)	0.533 (<0.01)	1.00
**Elevation**	-0.176 (0.03)	-0.163 (0.04)	-0.144 (0.08)	0.61	-0.160 (0.03)	-0.130 (0.06)	-0.130 (0.07)	0.63
***T***_**mean**_	-0.193 (0.21)		-0.178 (0.16)	0.54	-0.320 (0.02)	-0.194 (0.01)	-0.239 (0.04)	0.86
***T***_**seasonality**_	-0.128 (0.46)		-0.073 (0.65)	0.35	-0.216 (0.18)		-0.108 (0.53)	0.33
***P***_**ann**_	-0.132 (0.14)	-0.189 (0.01)	-0.156 (0.07)	0.63	-0.172 (0.04)	-0.156 (0.05)	-0.171 (0.05)	0.71
***P***_**seasonality**_	0.223 (0.01)	0.186 (0.02)	0.204 (0.02)	0.87	0.142 (0.07)	0.187 (0.01)	0.171 (0.02)	0.81
**Human impact**	-0.166 (0.06)	-0.143 (0.07)	-0.155 (0.07)	0.66	-0.047 (0.55)		-0.024 (0.76)	0.22
***T***_**velocity**_	-0.103 (0.32)	-0.164 (0.04)	-0.148 (0.12)	0.57	-0.247 (0.01)	-0.256 (<0.01)	-0.243 (0.01)	0.94
***P***_**velocity**_	0.000 (1.00)		-0.050 (0.66)	0.30	0.164 (0.18)		0.124 (0.32)	0.34
**Moran’s *I***	0.22 (0.01)	0.25 (<0.01)			-0.16 (0.47)	-0.15 (0.49)		
***R***^**2**^	0.43 (<0.01)	0.42 (<0.01)			0.65 (<0.01)	0.64 (<0.01)		

See Table 1 for description of table elements.

**Fig 3:**
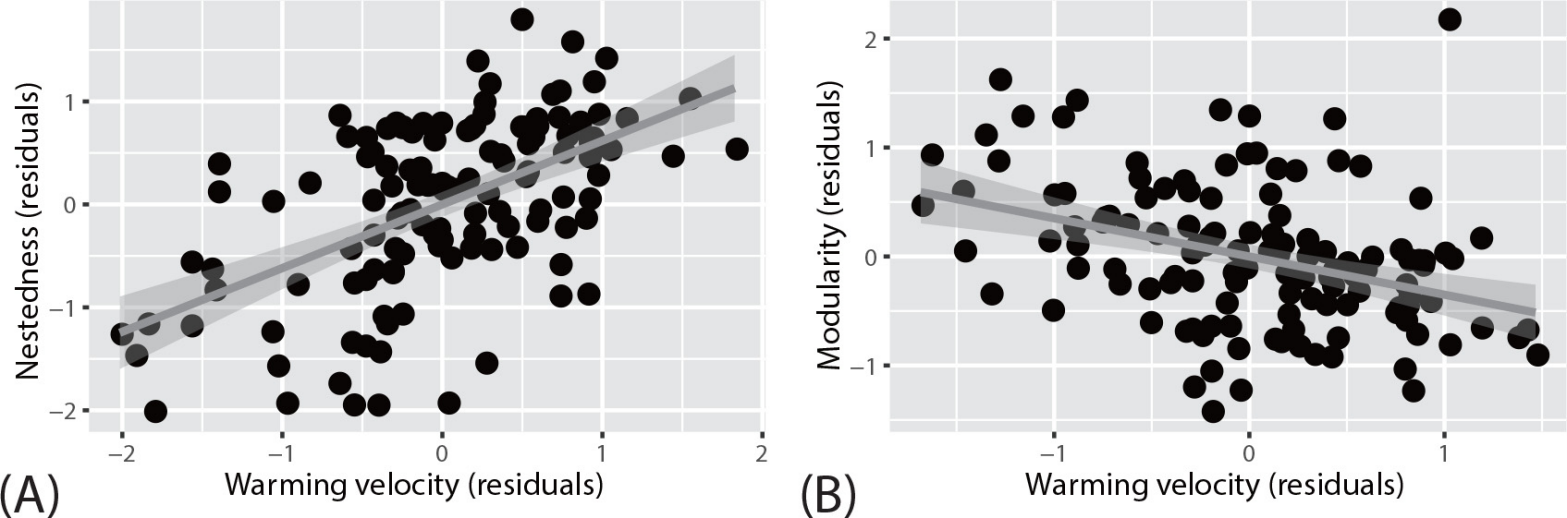
Scatter plots of a network parameter (residuals) versus temperature-change (i.e., warming) velocity (residuals) in food webs. Nestedness (A) and modularity (B).

Food-web structure was found to be associated with temperature-change velocity. In particular, nestedness increased as velocity increased (Fig 3A and Table 3); moreover, modularity decreased in response to velocity when spatial-autocorrelation effects were removed (Fig 3B and Table 4). No relationships were found between human impacts and either nestedness or modularity.

Current climate also affected both nestedness and modularity (Tables 3 and 4); a positive association was found between mean annual temperature and nestedness, whereas a negative association was detected between mean annual temperature and modularity. A positive correlation between modularity and precipitation seasonality was also observed, a result consistent with that of previous research [29].

### Seed-dispersal network modularity decreased with temperature-change velocity

In order to make comparisons with the results of a previous study [32], we re-examined the effects of human impacts and temperature-change velocity on nestedness and modularity in seed-dispersal networks, as the dataset used in this study differed slightly from that used in the earlier work.

**Fig 4:**
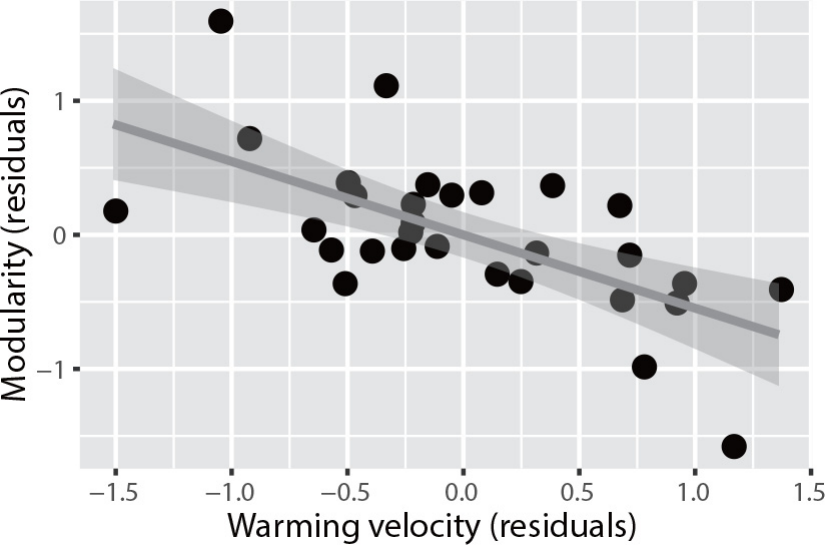
Scatter plot of modularity (residuals) versus temperature-change velocity (residuals).

We found no relationship between nestedness and human impacts/temperature-change velocity; moreover, we did not identify any variable explaining nestedness (S8 Table). On the other hand, there was a negative correlation between modularity and temperature-change velocity (Fig 4), in addition to elevation (Table 5), but no association was observed between human impacts and modularity. These results support the conclusion that environmental changes affect network structure in pollination [31] and seed-dispersal [32] networks; however, they were inconsistent with the results of previous research [32] in which a relationship was detected between human impacts and both nestedness and modularity.

**Table 5:** Influences of explanatory variables on modularity in seed-dispersal networks. See Table 1 for description of table elements.

Variables	OLS				SEVM			
	Estimate (full)	Estimate (best)	Estimate (averaged)	Importance	Estimate (full)	Estimate (best)	Estimate (averaged)	Importance
**#Species**	0.624 (<0.01)	0.663 (<0.01)	0.653 (<0.01)	1.00	0.575 (<0.01)	0.625 (<0.01)	0.604 (<0.01)	1.00
**Elevation**	-0.685 (0.03)		-0.244 (0.26)	0.37	-0.572 (0.07)	-0.466 (<0.01)	-0.442 (0.01)	0.84
***T***_**mean**_	-1.045 (0.06)		-0.002 (0.99)	0.22	-0.417 (0.43)		0.110 (0.70)	0.16
***T***_**seasonality**_	-0.806 (0.09)		-0.138 (0.59)	0.22	-0.492 (0.23)		-0.263 (0.16)	0.32
***P***_**ann**_	0.030 (0.86)		0.025 (0.88)	0.17	0.011 (0.94)		0.031 (0.83)	0.09
***P***_**seasonality**_	0.226 (0.26)		0.119 (0.44)	0.23	0.036 (0.84)		-0.029 (0.82)	0.09
**Human impact**	-0.018 (0.90)		-0.040 (0.79)	0.17	-0.020 (0.89)		-0.052 (0.68)	0.09
***T***_**velocity**_	-0.741 (<0.01)	-0.295 (0.03)	-0.365 (0.04)	0.81	-0.683 (0.01)	-0.664 (<0.01)	-0.588 (<0.01)	0.97
***P***_**velocity**_	0.255 (0.25)		0.099 (0.55)	0.21	0.059 (0.77)		-0.075 (0.55)	0.11
**Moran’s *I***	0.08 (0.14)	0.12 (0.23)			-0.60 (0.80)	-0.50 (0.85)		
***R***^**2**^	0.66 (<0.01)	0.54 (<0.01)			0.83 (<0.01)	0.80 (<0.01)		

See Table 1 for description of table elements.

## Discussion

Here, we examined the non-random structural patterns (i.e., nestedness and modularity) of ecological networks based on data collected from several global datasets, and demonstrated that human impacts and climate change affect nestedness and modularity in a range of ecological networks, including food-web, pollination, and seed-dispersal networks. In pollination networks, human impacts were positively correlated with nestedness and negatively correlated with modularity, and modularity declined with temperature-change velocity in seed-dispersal networks. In theory [5], an increase in nestedness and/or decrease in modularity improves ecosystem stability; thus, these results are an indication that mutualistic networks form in such a way as to enhance ecosystem stability against environmental changes or perturbations. Moreover, nestedness increased and modularity decreased in response to temperature-change velocity in food-web networks. Unlike in mutualistic networks, however, decreasing nestedness and/or increasing modularity enhance ecosystem stability in antagonistic networks (i.e., food webs); as such, our results suggest that food-web stability decreases in response to environmental changes.

Additional research is required, however. For example, we estimated climate-change velocities based on the difference between the current and last glacial maximum climate conditions (Materials and Methods), as was done in previous studies [28,31,32]. However, the time-scale of these velocities may be too long in terms of ecological-network assemblies; a possible reason was discussed in a previous study [31], but briefly, this may be because of the most important climatic shift in the Quaternary (past 2.6 million yr). In particular, glacial cold maxima and warm interglacials were periodically repeated during this period, and the most recent, and one of the strongest, climatic shift occurred between last glacial maximum (21,000 yr bp) and the present. This recent shift has been shown to have influenced geographical patterns of species endemism, for example [35], suggesting that species composition (and ecological-network assemblies, as a result) are more unstable in areas that have experienced larger climatic shifts. However, it is also important to consider short-range climate-change velocity. In this context, a new index [36] of the velocity of temperature change, derived from spatial gradients and multi-model ensemble forecasts of rates of temperature increase over the 21^st^ century, may be useful.

Alternative hypotheses must also be considered, especially in regard to the relationship between current climate and ecological networks. In food-web networks, for example, modularity increased with precipitation seasonality; given that climate seasonality can be considered as an environmental perturbation [29], the observed associations suggest that food webs vary to increase ecosystem stability against climate seasonality, consistent with predictions. A number of food-web networks included in this study derived from freshwater and estuarine areas; thus, that precipitation seasonality has an effect on network structure may be a reasonable supposition [29]. In short, these results imply that ecological networks may be generally adapted to changing environmental conditions.

The results presented here are at least somewhat inconsistent with those of previous research on mutualistic networks. For example, one study [32] reported an association between nestedness/modularity and human impacts in seed-dispersal networks, whereas no relationship was observed here. The results of another study [31] indicated that nestedness and modularity are associated with temperature-change velocity in pollination networks, but again, no such relationships were observed in this study (Tables 1 and 2). These discrepancies may be due to the different datasets used in this study and those used in previous studies; for instance, the dataset on pollination used in our study (*n* = 62) is larger than that used in a previous study [31] (*n* = 54); moreover, the similarity of the datasets was low [Jaccard index (JI) of approximately 0.4 (35/81)]. Although the number of seed-dispersal networks is almost similar between this study (*n* = 30) and the previous study [32] (*n* = 34), the similarity of the datasets was low [JI ≈ 0.5 (21/43)] because the previous study included unpublished data. In addition, the discrepancy may also be partially attributed to methodological differences. For example, the previous study [31] did not consider climate seasonality and human impacts, and was based on a unipartite version of modularity despite pollination networks traditionally represented as bipartite networks. Moreover, they did not consider the standardization of nestedness (NODF), although the *Z*-score (i.e., standardization) allows comparisons of networks with different levels of connectivity (i.e., connectivity or matrix fill strongly affects NODF) [37].

As pointed out in our earlier study [29], the conclusions we reached here are limited to binary (i.e., unweighted) networks. However, it is also important to consider weighted network analysis, as a different conclusion may be derived from comparisons between weighted networks and binary networks. For example, nestedness is statistically significant in binary networks, but not in weighted networks [38], and temperature seasonality was correlated with the weighted version of modularity, but not with the binary version of modularity [28]. Nevertheless, binary networks were considered in this study because the datasets we used include numerous binary data (Materials and Methods). Moreover, the definition of interaction weight is not uniform throughout the ecological-network datasets. The interaction weight assigned to a given species pair is based on the number of contacts they share; however, the weight need to be corrected (or normalized) for factors such as sampling effort and species abundance. Such normalization methods differ among studies. Therefore, we assumed a binary network approach to represent all ecological networks in order to avoid issues resulting from these variations.

Definitions for nestedness and modularity also vary, although here we focused on NODF and *M* for ease of comparison to previous studies. For nestedness, one previous study [38] used a spectral graph approach, and proposed the largest eigenvalue of a community matrix (mutualistic networks) for measuring nestedness, instead of using NODF-like nestedness indices. Moreover, the heterogeneity of degree distributions was shown to dominantly determine nestedness [39], network measures that may be useful as alternative indices for nestedness. The definition of modularity used in this study and in many previous studies is well known to have resolution-limitation problems [40,41]. We avoided this issue as much as possible by using a simulated-annealing algorithm, according to [41]; however, the conclusions reached in this study regarding network modularity are unavoidably limited. Although network modularity is useful for revealing functional architecture in ecological networks [42], the modules detected based on network topology may be inconsistent with biologically functional modules because the link weight and overlap of functional modules that are observed in real-world networks were not included in this study. To minimize these restrictions, module detection methods for weighted networks [28] and overlapping communities (e.g., [43,44]) may be useful because they more accurately predict functional modules.

As mentioned in our previous study [29], our analysis has several limitations, as do many other analyses of ecological networks. For example, we examined antagonistic (i.e., food webs) and mutualistic networks separately despite a mixture of interaction types (i.e., antagonistic interactions and mutualistic interactions) being more representative of real-world ecosystems and thus ecosystem stability [3,4]. Therefore, measurements of network parameters for multiple network types need to be considered in the future in order to evaluate ecological networks under more realistic conditions. In particular, multiple network analysis, which is commonly used in social-network research, can take into consideration many different types of links [45].

It is possible that sampling effort affects nestedness and modularity when considering the species–area relationship [46], which states that the number of observed species increases with the observed area. In this study, we could not obtain the relevant information on sampling effort because it is not always clearly delineated in the literature. However, this limitation poses little problem because the effect of species number was removed from the statistical analysis, and earlier work [27] suggested that nestedness and modularity are mostly independent of sampling effort (observation area and observation time). In addition, we did not consider the effects of phylogenetic signals because species descriptions in the networks are partially unknown or ambiguous. However, the absence of phylogenetic signals is unlikely to have a significant effect, as several studies have reported that phylogenetic signals are weak in ecological networks [28,47]. Moreover, a restricted understanding of interspecific reactions (i.e., missing links) is a more serious limitation.

To avoid these limitations, larger-scale and more highly normalized databases should be constructed, and it is especially important that data on weighted networks be expanded. In this context, data sharing [48] may be important.

Although our conclusions must necessarily be considered preliminary due to these limitations, they may enhance our understanding of the effects of environmental change on ecological communities.

## Materials and Methods

### Construction of ecological networks

The construction of the ecological networks used here was based on the procedures established in our previous study [29]. Briefly, we used the GlobalWeb database [9] (www.globalwebdb.com) for data on food webs, whereas the supporting online material in [49], the Interaction Web DataBase (www.nceas.ucsb.edu/interactionweb/), and the Web-of-Life Database (www.web-of-life.es) were used for data on pollination (plant–pollinator) and seed-dispersal (plant–seed disperser) networks. After removing duplications, we extracted network, observation sites (i.e., latitude and longitude), and geographic (i.e., climate and human impact) data that were available in the literature and databases, obtaining in total 126 food-web (S1 Table), 62 pollination (S2 Table), and 30 seed-dispersal (S3 Table) networks.

Food webs were represented as directed versions of unipartite networks because prey–predator relationships are directed [9,26,50], whereas mutualistic networks (i.e., pollination and seed-dispersal networks) were represented as bipartite networks because mutualistic links are only drawn between two types of organisms (i.e., plants and animals) [10,11,27]. These networks were represented as binary networks because the literature and databases we referenced included numerous binary data: ~82% (103/126) of food-web networks, ~71% (44/62) of pollination networks, and ~53% (16/30) of seed-dispersal networks were binary.

### Climatic parameters, elevation, human impact, and climate-change velocities

Following the procedures of our previous study [29], we downloaded climate data for the latitudes and longitudes of identified observation sites at a spatial resolution of 2.5 min of a degree (i.e., ~ 5 km × 5 km = 25 km^2^) from the WorldClim database (version 1.4, release 3) [51] (www.worldclim.org) using R version 3.2.0 (www.R-project.org) and an R-package *raster* version 2.3–40. These included annual mean temperature (*T*_mean_) (× 10°C), temperature seasonality (standard deviation) (*T*_seasonality_), annual precipitation (*P*_ann_) (mm), and precipitation, or rainfall seasonality (coefficient of variation) (*P*_seasonality_). Elevations or altitudes (m) were extracted using the Google Elevation Application Programming Interface (developers.google.com/maps/documentation/elevation/).

For human impacts, we used the human footprint (HF, ranging from 0–100) score from the global human footprint dataset compiled by the Last of the Wild Project (version 2) [52]. The HF score is provided with a spatial resolution of 1-km grid cells. According to the descriptions in the Last of the Wild database (sedac.ciesin.columbia.edu/data/collection/wildareas-v2/methods), HF scores were calculated by normalizing the human influence index, defined as the sum of eight human activity-related variables: human population density, human land use and infrastructure (built-up areas, nighttime lights, land use/land cover), and human access (coastlines, roads, railroads, navigable rivers).

As with previous studies [32,35], we considered two climate-change velocities: temperature-change velocity (*T*_velocity_) and precipitation-change velocity (*P*_velocity_). Climate-change velocity is defined as the temporal climate gradient divided by the spatial climate gradient, with the temporal gradient in turn defined as the absolute difference between current and Last Glacial Maximum (LGM) climate conditions. We used the CCSM3 model-based LGM climate conditions (i.e., *T*_mean_ and *P*_ann_), with a spatial resolution of 2.5 min of a degree, from the WorldClim database (www.worldclim.org/past). The spatial gradient was calculated as the local slope of the current climate surface at the study site; we obtained the local slopes of *T*_mean_ and *P*_ann_ using the function *terrain* (with the option *neighbors = 4*) in the R package *raster*.

### Nestedness and modularity

Calculations of nestedness and modularity were performed using methods described in our previous study [29]. We used NODF scores [37] for evaluating nestedness because, unlike other definitions of nestedness, NODF does not have an overestimation problem [37]. The function *nestednodf* in the R package *vegan* version 2.3–0 was used for calculating NODF. When calculating NODF in food webs, we considered the food webs to be resource–consumer bipartite networks, in the same manner as in a previous study [10]. To measure modularity, a modularity score *M* was calculated (reviewed in [41]); specifically, we used the BIPARTMOD software [53] (seeslab.info/downloads/bipartite-modularity/) for calculating the modularity (*M*) of directed networks and bipartite networks because the BIPARTMOD software finds the maximum *M* based on simulated annealing in order to minimize the resolution-limit problem in community detection [40,41].

NODF and *M* were standardized as *Z*-scores to allow comparisons among matrices and to evaluate statistical significance, in accordance with previous studies [27,32,54]. The *Z*-score of a measure *X* was defined as *Z*_*X*_ = (*X*_real_−*X*_null_)/SD_null_, where *X*_real_ represents a network measure (i.e. NODF or *M*) of an empirical network, and *X*_null_ and SD_null_ represent the average network measure value and the standard deviation, respectively, obtained from 500 null-model networks. Degree-preserving randomization, a technique widely used in ecological-network analysis [27,28,32,38,54,55], was used to generate the null-model networks. Consideration of degree distributions is important for evaluating the statistical significance of network properties of real-world ecological networks because they influence both nestedness and modularity [17,56]. For food-web (i.e. unipartite directed networks) null-model networks, we used randomized networks generated from real-world food webs via an edge-switching algorithm [55]. This algorithm produces random networks by rewiring two randomly selected edges in the real-world network until the switching of all edges is completed, preserving the number of one-way incoming edges, one-way outgoing edges, and mutual edges for each node (see the supporting online material in [55] for details). For degree preserving, one-way edges and mutual edges are considered separately when switching two edges; for instance, consider two edges, A→B and C→D, where the letters and arrows represent nodes and one-way edges, respectively. Through this edge-switching algorithm, the edges A→D and C→B are obtained if the switching generates no multiple edges or self-edges. For mutual edges, two edges are switched through a similar procedure, with A↔D and C↔B generated from A↔B and C↔D. For pollination and seed-dispersal networks (i.e. bipartite networks), the null model II [10] was used, which generates random bipartite networks with degree distributions that are similar to those of empirical networks. In the null model, the probability that a plant connects to an animal is proportional to the product of node degrees for a given plant or animal.

### Statistical analyses

To evaluate the contribution of each variable (or factor) to a network measure, we conducted regression analyses using R software. The OLS regression was first considered, for which we constructed full models encompassing all explanatory variables (*T*_mean_, *T*_seasonality_, *P*_ann_, *P*_seasonality_, elevation, human impact (HF score), *T*_velocity_, and *P*_velocity_), and selected the best model based on the sample-size-corrected version of Akaike information criterion (AICc) values, using the R package *MuMIn* version 1.15.6. In accordance with previous studies [31,32], we also considered species richness, or the number of species *S* (i.e., the number of nodes in ecological networks), for two reasons: first, species richness may affect network measures, and second we intended to identify network patterns beyond species richness. *T*_velocity_ and *P*_velocity_ were log-transformed for all analyses. We estimated the relative importance of each explanatory variable by summing the weights of AIC across all models that consisted of a given set of variables [57] using the function *importance* in the R package *MuMIn*. We also adopted a model-averaging approach [31], from which we obtained the averaged models in the top 95% confidence set of models using the *model*.*avg* function in the R package *MuMIn*. A global Moran’s test was performed to evaluate spatial autocorrelation in the regression residuals using the function *lm*.*morantest*.*exact* in the R package *spdep* version 0.6–4.

Given that spatial autocorrelation was performed for most OLS regression models (i.e., *p* < 0.05, Moran’s test), we next considered a SEVM modeling approach to remove spatial autocorrelation in the regression residuals [31,57,58]. Specifically, we adopted the Moran eigenvector approach using the function *SpatialFiltering* in the R package *spdep*. As with the OLS regression analyses, we constructed full models and then selected the best model based on AICc values. The spatial filter was fixed in the model-selection procedures [57]. We also obtained the averaged models, and estimated the relative importance of each explanatory variable in the SEVM modeling. The residuals of the explanatory variables and network parameters were calculated according to the SEVM modeling approach-based best models.

The contribution (i.e., non-zero estimate) of each explanatory variable to a network parameter was completed when the associated *p*-value was less than 0.05.

## Supporting Information

S1 Table List of food webs. This table includes the network ID, network type (i.e., binary or weighted), reference, nestedness (standardized NODF), modularity (standardized *M*), the number of species (#Species), the number of links (#Links), climatic and geographic parameters [i.e., longitude, latitude, elevation, mean annual temperature (*T*_mean_), temperature seasonality (*T*_seasonality_), annual precipitation (*P*_ann_), precipitation seasonality (*P*_seasonality_), human impact, temperature-change velocity (*T*_velocity_), and precipitation-change velocity (*P*_velocity_)].
(CSV)

S2 Table List of pollination networks. See S1 Table for description of table elements; note however that this table includes the number of plants (#Plants) and the number of animals (#Animals) instead of the number of species.
(CSV)

S3 Table List of seed-dispersal networks. See S1 Table for description of table elements; note however that this table includes the number of plants (#Plants) and the number of animals (#Animals) instead of the number of species.
(CSV)

S4 Table Influence of explanatory variables on nestedness in mainland pollination networks. See Table 1 for description of table elements.
(XLS)

S5 Table Influence of explanatory variables on nestedness in island pollination networks. See Table 1 for description of table elements.
(XLS)

S6 Table Influence of explanatory variables on modularity in pollination mainland networks. See Table 1 for description of table elements.
(XLS)

S7 Table Influence of explanatory variables on modularity in island mainland networks. See Table 1 for description of table elements.
(XLS)

S8 Table Influence of explanatory variables on nestedness in seed-dispersal networks. See Table 1 for description of table elements.
(XLS)
